# Relationship between drug burden and physical and cognitive functions in a sample of nursing home patients with dementia

**DOI:** 10.1007/s00228-017-2319-y

**Published:** 2017-09-18

**Authors:** L. M. J. Sanders, T. Hortobágyi, G. van Staveren, K. Taxis, F. Boersma, H. C. Klein, W. J. R. Bossers, C. G. Blankevoort, E. J. A. Scherder, E. A. Van der Zee, M. J. G. van Heuvelen

**Affiliations:** 1 Center for Human Movement Sciences, University Medical Center Groningen, University of Groningen, Antonius Deusinglaan 1, 9713 AV Groningen, The Netherlands; 2 Emergency Department, Medical Center Leeuwarden, Henri Dunantweg 2, 8934 AD Leeuwarden, The Netherlands; 3 Groningen Research Institute of Pharmacy (GRIP), University Medical Center Groningen, University of Groningen, Antonius Deusinglaan 1, 9713 AV Groningen, The Netherlands; 4 Department of General Practice, Elderly Care Medicine, University Medical Center Groningen, University of Groningen, Antonius Deusinglaan 1, 9713 AV Groningen, The Netherlands; 5 Department of Psychiatry, University Medical Center Groningen, University of Groningen, Hanzeplein 1, 9713 GZ Groningen, The Netherlands; 6 Department of Geriatric Psychiatry, Lentis, Hereweg 80, 9725 AG Groningen, The Netherlands; 7 Department of Clinical Neuropsychology, VU University Amsterdam, Van der Boechorstraat 1, 1081 BT Amsterdam, The Netherlands; 8 Groningen Institute for Evolutionary Life Sciences (GELIFES), University of Groningen, Nijenborgh 7, 9747 AG Groningen, The Netherlands

**Keywords:** Drug Burden Index, Dementia, Inappropriate prescribing, Nursing home facilities, Medication

## Abstract

**Purpose:**

The Drug Burden Index (DBI) is a tool to quantify the anticholinergic and sedative load of drugs. Establishing functional correlates of the DBI could optimize drug prescribing in patients with dementia. In this cross-sectional study, we determined the relationship between DBI and cognitive and physical functions in a sample of patients with dementia.

**Methods:**

Using performance-based tests, we measured physical and cognitive functions in 140 nursing home patients aged over 70 with all-cause dementia. We also determined anticholinergic DBI (AChDBI) and sedative DBI (SDBI) separately and in combination as total drug burden (TDB).

**Results:**

Nearly one half of patients (48%) used at least one DBI-contributing drug. In 33% of the patients, drug burden was moderate (0 < TDB < 1) whereas in 15%, drug burden was high (TDB ≥ 1). Multivariate models yielded no associations between TDB, AChDBI, and SDBI, and physical or cognitive function (all *p* > 0.05).

**Conclusions:**

A lack of association between drug burden and physical or cognitive function in this sample of patients with dementia could imply that drug prescribing is more optimal for patients with dementia compared with healthy older populations. However, such an interpretation of the data warrants scrutiny as several dementia-related factors may confound the results of the study.

**Electronic supplementary material:**

The online version of this article (10.1007/s00228-017-2319-y) contains supplementary material, which is available to authorized users.

## Introduction

With the world population progressively growing older, the number of older adults with dementia increases. Globally, 47.5 million people suffer from dementia, resulting in a health care expenditure of $600 billion [1]. Cognitive and physical abilities of patients with dementia decline steadily, compromising daily function and independence and requiring approximately one half of patients to move to a nursing home (NH) and receive assistance [2]. The prevalence of comorbidities among patients with dementia is high [3]. Depending on the study, 43 to 92% of dementia patients are exposed to polypharmacy, i.e., the concurrent use of five or more medications from different drug categories [4–7], which can result in serious adverse drug reactions (ADRs) such as cognitive impairment, functional decline, and an increase in risk of falls and fractures [8, 9]. To minimize suboptimal drug use by older adults, the Beers criteria categorize inappropriate drugs that patients should use with caution or avoid [10]. Even though anticholinergic and sedative psychotropic drugs have especially high risks to cause adverse effects, it is estimated that 23 to 47% of dementia patients take at least one anticholinergic or sedative drug [11–13].

Several tools exist to quantify anticholinergic drug burden in older adults, but the agreement between tools is limited [14]. The Drug Burden Index (DBI) has been identified as an appropriate tool to quantify anticholinergic and sedative drug burden in older adults [15–17]. In community-dwelling older adults, a higher DBI correlates with low physical function [18–21], high fall rate [22], difficulty in performing activities of daily living (ADLs) [20, 21], mortality, and hospitalization [23]. However, the evidence is mixed regarding the relationship between DBI and cognitive function [15, 24, 25]. In dementia patients, a higher DBI correlates with low self-reported health-related quality of life [7] and a high risk of hospitalization and mortality [23]. As far as we know, the relationship between DBI and physical and cognitive functions has not yet been examined in patients with dementia. To minimize suboptimal drug prescribing for patients with dementia, it is necessary to establish the functional associations of the DBI in this patient group.

The purpose of the present cross-sectional study was to determine the relationship between DBI and cognitive and physical functions in patients with all-cause dementia. We quantified the relationship between anticholinergic DBI (AChDBI), sedative DBI (SDBI), and total drug burden (TDB = AChDBI + SDBI), and physical and cognitive functions. We hypothesized that AChDBI and SDBI individually and in combination in the form of TDB are inversely associated with physical and cognitive functions.

## Methods

### Design

This study combined data from a Dutch cross-sectional (Nederlands Trial Register (NTR)1230, 74 participants, data collected between 2004 and 2007) study and baseline data from a related Dutch intervention study (NTR2269, 66 participants, collected between 2010 and 2014). Each participant or a legal representative signed an informed consent approved by the University Medical Ethical Committee. The studies were conducted in accordance with the principles of the Declaration of Helsinki (64th amendment).

### Sample and procedures

The current sample consisted of 140 NH residents over age 70 with a dementia diagnosis. The two studies differed minimally in terms of inclusion and exclusion criteria, resulting in the following overall inclusion criteria for the total sample: age > 70, a physician-diagnosed dementia reported in the medical chart, the ability to walk short distances without a walking aid, and an Mini Mental State Examination (MMSE) score ≥ 10 and ≤ 24 (indicating mild to moderate dementia). Multiple disease-related exclusion criteria were used for safety reasons (see NTR files 1230 and 2269, Appendix 1). Appendix 1 also describes the recruitment procedures. Trained research assistants recorded data on sociodemographic factors (age, gender, level of education) and cognitive and physical abilities. To minimize test burden, the assessor performed the cognitive and physical assessments in two separate sessions within 7 days. The researchers extracted data on medical conditions and medication use for all participants from the nursing homes’ medical files. Medication data comprised a record of all medications taken by the participant at the time of assessment and the subsequent dose, frequency, and method of administration. We excluded medication *as needed*, topical ointments, lubricating eye drops, and over-the-counter medications from the current analyses.

### Measurements

#### Medication assessment

To code the drugs, we used the Anatomical Therapeutic Chemical (ATC) classification system, as recommended by the World Health Organization [26]. We quantified the total anticholinergic and sedative load with the DBI and calculated TDB as follows [15]:

1. TDB = AChDBI + SDBI

where AChDBI and SDBI represent, respectively, total anticholinergic and sedative load. We determined sedative and/or anticholinergic load for each drug and summed up as follows:

(2) AChDBI or SDBI = Σ *D*_*i*_ / (*δ*_*i*_ + *D*_*i*_)

where *D*_*i*_ represents the daily dose taken by the participant and *δ* represents the recommended minimum daily dose of the drugs with an anticholinergic or sedative load, respectively. The recommended minimum daily dose was specified for each drug (i) based on the lowest minimum oral dose that is prescribed for any common medical indication in older adults.

#### Classification of anticholinergic and sedative drugs

**Fig. 1:**
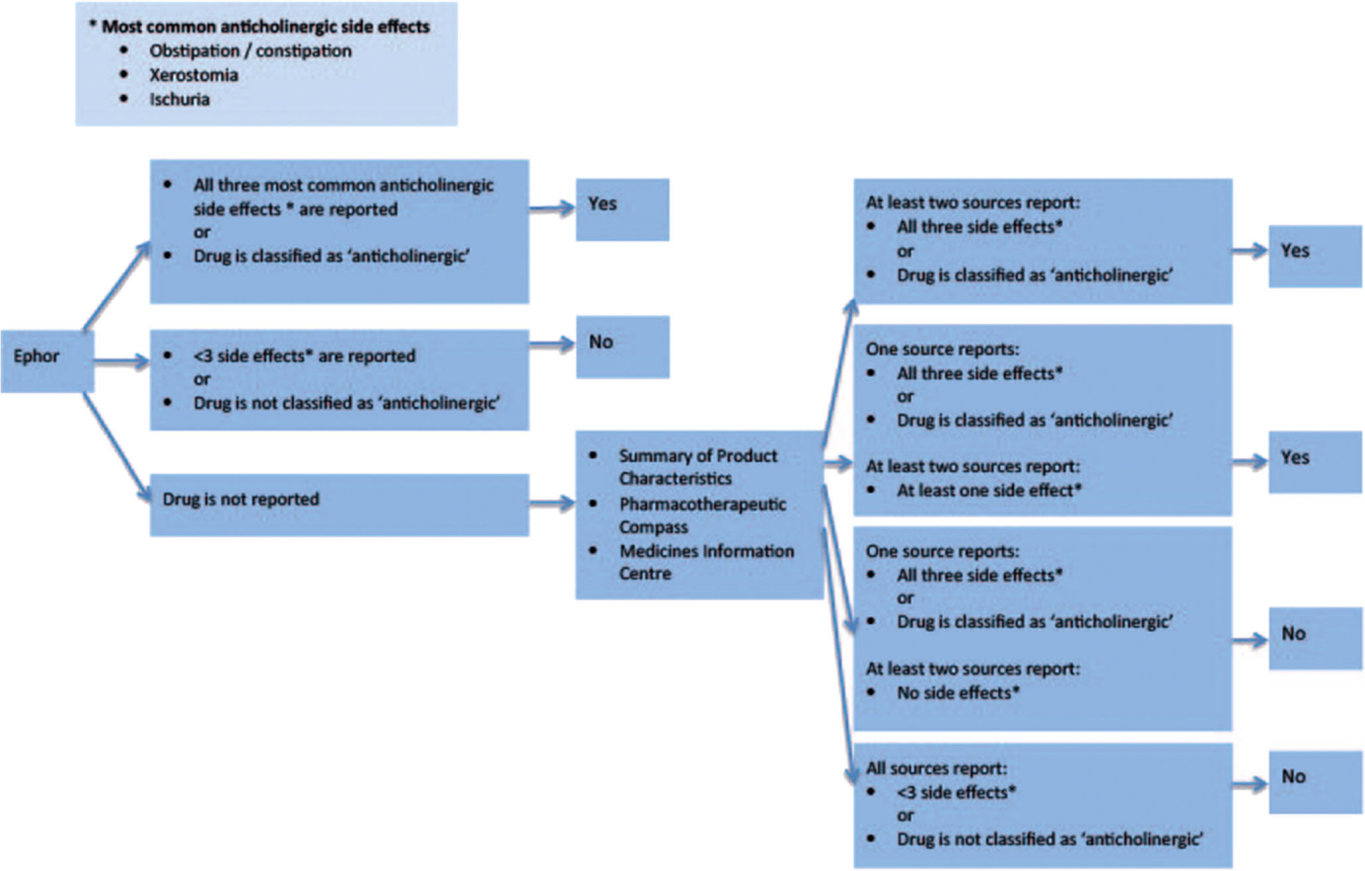
Classification process of drug as anticholinergic

**Fig. 2:**
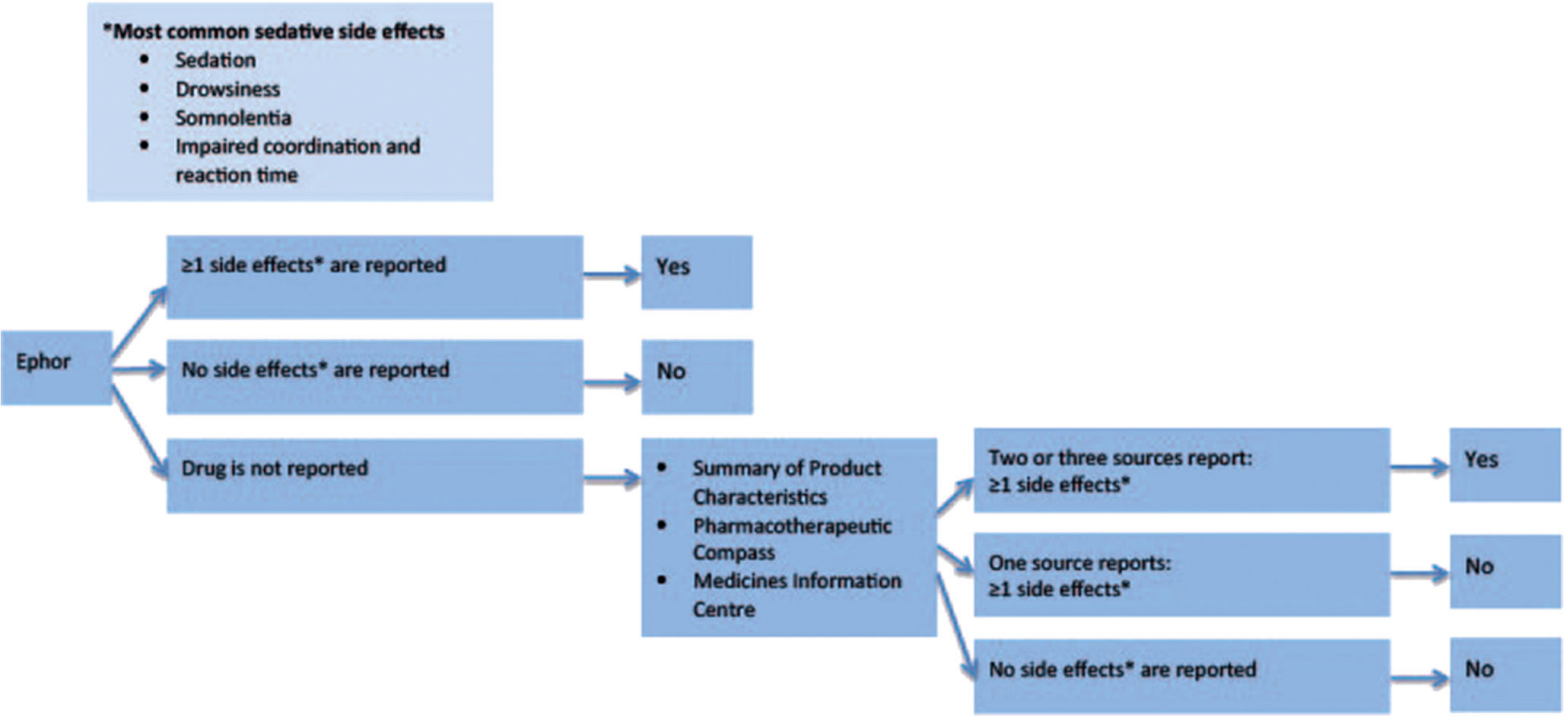
Classification process of drug as sedative

To determine if a drug had sedative and/or anticholinergic effects, the Expertise Centre Pharmacotherapy in Older persons (Ephor) [27] was consulted. Figures 1 and 2 show the decision tree for the classification process. A drug was classified as anticholinergic when the three most common anticholinergic side effects (obstipation/constipation, xerostomia, and urinary retention) were described in Ephor or in at least two of the remaining sources: the Summary of Product Characteristics (SmPC [28], the Pharmacotherapeutic Compass [29], and the Medicines Information Centre of the Royal Dutch Pharmacists Association [30]. If all three anticholinergic side effects were mentioned in one of the remaining sources and at least one side effect was mentioned in the two other remaining sources, the drug was also classified as anticholinergic. A drug was classified as sedative when either sedation, drowsiness, somnolence, or impaired coordination and reaction time were listed as side effects in Ephor or at least two of the remaining sources. If a drug was known to have both anticholinergic and sedative effects, it was classified as anticholinergic [15].

#### Polypharmacy

Polypharmacy and excessive polypharmacy were defined as the concurrent use of five to nine and more than nine drugs, respectively [4].

#### Non-DBI-contributing drugs

Other than inclusion in polypharmacy measures, we excluded all non-DBI-contributing drugs from the current analyses.

#### Comorbidities

We quantified comorbidity based on the Functional Comorbidity Index (FCI-18) [31] (Appendix 3). The FCI comprises 18 medical conditions that negatively impact physical function. The presence or absence of each condition is listed. A higher score represents a greater number of comorbidities.

#### Functional outcomes

##### Motor function

To characterize motor function, we used several performance-based tests that are frequently used for patients with dementia [32]. Functional mobility was quantified with the Six Meter Walk Test (meters per second) [33], Timed Up&Go (seconds) [34], and 30-seconds Sit to Stand (number of correct attempts) [35]. Balance was measured with the Frailty and Injuries Cooperative Studies of Intervention Techniques subtest 4 (FICSIT-4) [36] and Figure of Eight (seconds) [37]. Grip strength (kg) was assessed using a Jamar^©^ hand dynamometer.

##### Cognitive function

We employed frequently used neuropsychological tests [32] to quantify cognitive function, including global cognitive function (Mini Mental State Examination) [38]; verbal memory (Eight Word Task immediate recall and recognition) [39]; verbal working memory (Digit Span Forward and Backward) [40]; visual memory (Visual Memory Span Forward and Backward) [40]; Rivermead Behavioural Memory Test (RBMT) Faces and Pictures [41]; abstract reasoning (Groninger Intelligence Test (GIT) incomplete figures) [42]; and basic information processing speed (STROOP word card, adapted 45-s version) [43]. We determined the number of correct responses for all tests as an outcome measure. For all tasks, higher scores indicate a better performance.

### Statistical analyses

We used SPSS Statistics 23.0 (IBM, Armonk, NY) to compute means and standard deviations (SDs) for motor and cognitive outcomes and to analyze the data. Scores on the Six Meter Walk Test, Timed Up&Go, and Figure of Eight were positively skewed and, thus, log10-transformed. We imputed missing data for cognitive (5.8% missing) and physical (6.0% missing) variables using the maximum likelihood-expectation maximization algorithm [44]. The scores on the cognitive and motor tests as well as sociodemographic factors and comorbidities were used as predictors for missing data completions.

We set the DBI as a categorical ordinal variable (DBI = 0, 0 < DBI < 1, DBI ≥ 1) [19]. Uncontrolled and controlled multivariate analyses (MANOVAs) were performed to assess any differences in functional outcomes between the DBI classes. The analyses were done separately for the cognitive and the physical performance scores, as well as for TDB, AChDBI, and SDBI values.

We identified potential confounders by determining the correlation between sociodemographic factors, comorbidity (FCI-18), and cognitive and physical outcomes. Potential confounders for cognitive outcomes were age, gender, and education. Additionally, FCI-18 was added to the models as a confounder. Potential confounders for physical outcomes included age, gender, education, use of walking aid, and additionally, FCI-18. We presented the non-transformed data for transformed variables. We used two-tailed tests, and statistical significance was set at *p* < 0.05.

## Results

Table 1 shows participants’ characteristics (*n* = 140, 78.6% female). The mean age was 85.1 ± 5.7 years. The most frequent dementia diagnoses were Alzheimer’s disease and/or vascular dementia (92.8% of all cases). The mean MMSE score was 16.2 ± 4.5, thus being indicative of moderate dementia. Polypharmacy (*n* = 68, 48.6%) and excessive polypharmacy (*n* = 24, 17.1%) were frequent. In total, 105 (75.0%) participants took one or more drugs with anticholinergic (*n* = 69, 49.3%) or sedative (*n* = 36, 25.7%) effects. Of these participants, 46 (32.9%) had a TDB value between 0 and 1 and 21 (15.0%) had a TDB value of ≥ 1. Five participants used a cholinesterase inhibitor (all rivastigmine). Of these five participants, only two had a TDB value > 0 (TDB = 0.6 and TDB = 2.5), thereby limiting the chance of prescribing cascades between anticholinergics and cholinesterase inhibitors in the current sample. The total number of drugs did not correlate with TDB, AChDBI, or SDBI. Dementia severity as measured by a MMSE score did not correlate with TDB, AChDBI, or SDBI (*r* = − 0.003, *r* = − 0.002, and *r* = − 0.002, respectively). Age inversely correlated with TDB (*r* = − 0.200, *p* = 0.018). Table 1 summarizes patient characteristics in the TDB subgroups. Appendix 2 lists the DBI-contributing drugs used in the sample. The most commonly used anticholinergic drug was the antidepressant citalopram (*n* = 16). Oxazepam, a benzodiazepine, was the most commonly used sedative drug (*n* = 12).

**Table 1:** Patient characteristics in the total sample (*N* = 140)

Characteristics	Value^a^	TDB = 0	0 < TDB < 1	TDB ≥ 1
*N* (% of total)	140 (100)	73 (52.1)	46 (32.9)	21 (15.0)
Age (years; mean, SD)	85.13 (5.69)	85.92 (5.71)	84.85 (5.38)	83.00 (5.90)
Gender (*N* women, % of total)	110 (78.6)	58 (79.5)	35 (76.1)	17 (81.0)
Education (*N*, % of total)				
1 = primary education only	29 (23.6)	14 (21.2)	12 (30.8)	3 (16.7)
2 = secondary lower education	77 (62.6)	42 (63.6)	22 (56.4)	13 (72.2)
3 = secondary higher education	17 (13.8)	10 (15.2)	5 (12.8)	2 (11.1)
Use of walking aid (*N*, % of total)				
No	64 (45.7)	35 (47.9)	22 (47.8)	10 (47.6)
Yes	70 (50.0)	38 (52.1)	24 (52.2)	11 (52.4)
Dementia diagnosis according to medical file (*N*, % of total)				
1 = Alzheimer’s disease (AD)	86 (61.4)	49 (67.1)	26 (56.6)	13 (61.9)
2 = vascular dementia (VD)	16 (11.4)	6 (8.2)	10 (21.8)	3 (14.3)
3 = mixed (AD + VD)	28 (20.0)	16 (21.9)	10 (21.7)	2 (9.5)
4 = dementia with Lewy bodies (DLB)	5 (3.6)	2 (2.7)	0 (0.0)	3 (14.3)
MMSE, mean (SD)	16.16 (4.5)	16.49 (0.53)	15.44 (0.66)	16.57 (0.98)
Total Drug Burden Index	0.43 (0.58)	0.00 (0.00)	0.61 (0.18)	1.55 (0.44)
Anticholinergic Drug Burden Index	0.27 (0.43)	0.00 (0.00)	0.40 (0.28)	0.92 (0.58)
Sedative Drug Burden Index	0.16 (0.33)	0.00 (0.00)	0.21 (0.30)	0.63 (0.46)
Total number of medications used (mean, SD)	5.71 (3.07)	5.64 (3.03)	5.67 (2.94)	6.05 (3.54)
Polypharmacy (*N*, % of total)				
No	48 (34.3)	36 (49.3)	11 (23.9)	1 (4.8)
Polypharmacy (5–9)	68 (48.6)	28 (38.4)	26 (56.5)	14 (66.7)
Excessive polypharmacy (> 9)	24 (17.1)	9 (12.3)	9 (19.6)	6 (28.6)
Functional Comorbidity Index total (mean, SD)	1.88 (1.52)	1.65 (1.36)	2.14 (1.52)	2.15 (1.69)

*TDB* total Drug Burden Index

^a^Data were missing for level of education (*n* = 17), use of walking aid (*n* = 6), dementia diagnosis (*n* = 5), and Functional Comorbidity Index total (*n* = 11)

### Relationship between DBI and physical function

The model not controlled for confounders revealed no group differences in measures of motor performance in the TDB subgroups (*F*(12,264) = 1.159, *p* = 0.313, Wilks’ *Λ* = 0.902, partial *η* ^2^ = 0.050). The same applies to physical performance in AChDBI (*F*(12,264) = 0.538, *p* = 0.889, Wilks’ *Λ* = 0.953, partial *η* ^2^ = 0.024) and SDBI (*F*(12,264) = 1.382, *p* = 0.174, Wilks’ *Λ* = 0.885, partial *η* ^2^ = 0.059) subgroups. After controlling the models for age, gender, education, and use of walking aid, there were no differences in physical outcomes between the subgroups TDB (*F*(12,220) = 1.063, *p* = 0.393, Wilks’ *Λ* = 0.893, partial *η* ^2^ = 0.055), AChDBI (*F*(12,220) = 0.766, *p* = 0.685, Wilks’ *Λ* = 0.921, partial *η* ^2^ = 0.040), or SDBI (*F*(12,220) = 1.121, *p* = 0.344, Wilks’ *Λ* = 0.888, partial *η* ^2^ = 0.058). When the FCI score is taken into account as an additional covariate, there were no group differences for TDB (*F*(12,206) = 1.114, *p* = 0.350, Wilks’ *Λ* = 0.882, partial *η* ^2^ = 0.061), AChDBI (*F*(12,206) = 0.726, *p* = 0.725, Wilks’ *Λ* = 0.920, partial *η* ^2^ = 0.041), and SDBI (*F*(12,206) = 1.303, *p* = 0.219, Wilks’ *Λ* = 0.864, partial *η* ^2^ = 0.071).

### Relationship between DBI and cognitive function

In the multivariate model not controlled for potential confounders, cognitive outcomes were not different for the TDB classes (*F*(22,254) = 1.191, *p* = 0.256, Wilks’ *Λ* = 0.822, partial *η* ^2^ = 0.093). We found equivocal results when the AChDBI (*F*(22,254) = 0.976, *p* = 0.495, Wilks’ *Λ* = 0.850, partial *η* ^2^ = 0.078) or the SDBI (*F*(22,254) = 1.005, *p* = 0.459, Wilks’ *Λ* = 0.846, partial *η* ^2^ = 0.080) were taken into account separately. After controlling for age, gender, and education, there were no differences in cognitive outcomes between the subgroups TDB (*F*(22,220) = 1.303, *p* = 0.171, Wilks’ *Λ* = 0.783, partial *η* ^2^ = 0.115), AChDBI (*F*(22,220) = 1.074, *p* = 0.377, Wilks’ *Λ* = 0.815, partial *η* ^2^ = 0.097), and SDBI (*F*(22,220) = 1.170, *p* = 0.277, Wilks’ *Λ* = 0.801, partial *η* ^2^ = 0.105). With FCI as an additional covariate, there were no differences in cognitive outcomes between the TDB (*F*(22,206) = 1.352, *p* = 0.142, Wilks’ *Λ* = 0.764, partial *η* ^2^ = 0.126), AChDBI (*F*(22,206) = 1.052, *p* = 0.403, Wilks’ *Λ* = 0.808, partial *η* ^2^ = 0.101), and SDBI (*F*(22,206) = 1.250, *p* = 0.210, Wilks’ *Λ* = 0.778, partial *η* ^2^ = 0.118) subgroups.

## Discussion

To our best knowledge, this is the first cross-sectional study to examine functional correlates of the DBI in a population of patients with mild-to-moderate all-cause dementia. We used a wide range of reliable and valid measurements to assess cognitive and physical functions. We found no multivariate relationships between DBI and cognitive and physical functions.

### Relationship between drug burden and physical function

In the present study, TDB did not correlate with physical function. We hypothesized that TDB would negatively correlate with physical function because anticholinergic and sedative drugs target central nervous system (CNS) functions that affect physical function, such as the gastrointestinal system and neuromuscular processes [21]. A lack of association between TDB and physical function in our study contrasts with data in cognitively healthy populations [18–20], possibly for two dementia-related potential reasons.

First, drug prescribing might be more optimal for patients with dementia compared with healthy older populations. For dementia patients, a larger emphasis may be placed upon tolerability and quality of life instead of treatment of symptoms and quantity of life, resulting in better-tailored drug prescribing [45]. Indeed, in our sample, less than half of patients used DBI drugs, which is a lower rate compared with the rate in a sample of healthier older adults [46]. Such a careful approach may become even more pronounced with older age, a hypothesis perhaps reflected by a negative relationship between age and TDB in our study. Not only the presence but also the severity of dementia may be related to more appropriate prescribing. However, dementia severity (as measured with MMSE) and DBI were unrelated in our study, confirming a lack of covariation between the odds of being prescribed inappropriate medication and dementia severity [6]. Thus, the presence rather than severity of dementia may be a better indicator of lower risk of suboptimal prescribing.

A second reason why our results showed no relationship between physical function and drug burden could be that numerous dementia-related factors that affect physical health might confound the relationship between TDB and physical function in patients with dementia. Dementia progression [47], age, poor health [48], sedentariness [49], adverse life events, and a decline in general well-being [50] all unfavorably affect physical function. The presence and manifestation of such factors may greatly vary in patients with dementia and thus increase variability within the sample. In combination, these factors might minimize the influence of DBI drugs on physical and cognitive function, nullifying a potential relation. However, this hypothesis of confounding factors is weakened by the results of a previous randomized controlled trial (RCT) that aimed to reduce anticholinergic exposure through a 12-week reduction intervention in institutionalized patients with dementia. The authors found that physical function as measured with the Barthel Index did not improve after a decrease in anticholinergic exposure after 12 weeks [51]. Considering that the randomized controlled nature of the study should minimize the influence of confounding variables, we cannot definitively conclude that confounding factors underlie the lack of relationship between drug burden and physical function.

Our findings qualitatively agree with data in hospitalized patients with multimorbidity, 43.5% of whom suffered from dementia. In these patients, the use of three or more psychotropic drugs was related to lower hand-grip strength in both hands, but not lower extremity muscle strength [52]. However, further comparisons of our sample with other dementia populations are difficult because the relationship between drug burden (DBI or other measures) and physical function in patients with dementia is understudied. Future research should improve our understanding of the relationship between physical function and drug burden in this patient group. Within such research, it is important to consider the care setting as being a determinant for more appropriate prescribing in patients with dementia. Our sample included patients with dementia in nursing homes, who may be at a lower risk of suboptimal prescribing compared with community-dwelling populations with or without cognitive impairment [23, 46]. Indeed, the use of DBI drugs declines by approximately 5% after NH admission [53, 54], although there is a paucity of data concerning DBI drug usage in specifically Dutch NHs. There may be four reasons why DBI drug prescribing may be more optimal after NH admission: (1) NH physicians may be less inclined to prescribe DBI drugs because they are specialized in the medical aspects of older patients compared with primary care physicians [54]; (2) NH staff can quickly recognize and address the adverse effects of DBI-contributing drugs; (3) behavioral disturbances are generally less medicalized in a NH versus home setting because behavior is interpreted in a broader, more accepting context and approached as such [55]; and (4) NH physicians, in particular, might recognize the diminished benefit of drugs in light of patients’ low functional level [56, 57]. However, the present study could not examine in more detail the impact of care setting on drug burden, as we studied only a NH population.

In addition to TDB, we hypothesized that higher AChDBI and SDBI separately correlated with lower physical function. Separate associations of AChDBI versus SDBI with physical function could arise from pharmacodynamic differences between these two drug classes: whereas anticholinergic drugs mainly target the cholinergic system involved in excitatory processes, sedative drugs generally influence inhibitory mechanisms by targeting levels of gamma-aminobutyric acid (GABA), although several DBI drugs target both systems (e.g., citalopram). In older women, anticholinergic compared with sedative drug burden (not measured with DBI) more strongly correlated with impaired balance, mobility, gait, chair stands, and grip strength [58]. Sedative burden was associated with impaired mobility and grip strength only. In contrast, Gnjidic et al. [21] reported that SDBI predicted poorer performance on measures of gait, balance, and grip strength in older men, whereas AChDBI was associated with weaker grip strength only. The difference between these studies might be explained by differences in sedative drug usage [58] or gender differences. However, contrasting with these studies, neither AChDBI nor SDBI correlated with physical function in our sample. The effects of anticholinergic and sedative drugs may be less discernible in dementia patients versus community-dwelling populations because amyloid β deposition in Alzheimer’s disease (AD) disrupts the excitatory/inhibitory balance system [59]. Consequently, DBI drugs that target the anticholinergic system may disrupt the GABA system as well (and vice versa). Thus, functional correlates of AChDBI/SDBI, if any, may be indiscernible in dementia patients.

### Relationship between drug burden and cognitive function

Contrary to our hypothesis, we found no association between TDB and cognitive function. Associations between higher drug burden and lower cognitive function in other populations can be explained by the detrimental effects of anticholinergic and sedative drugs on CNS processes involving vision, attention, sedation, and psychomotor speed [21]. The finding that higher anticholinergic burden was not related to lower cognitive function is similar to the results of a study in 224 community-dwelling patients with AD, where anticholinergic load was quantified with the Anticholinergic Burden scale [60], and in line with a study in patients with multimorbidity (43.5% dementia) showing that users of anticholinergic or sedative drugs did not have lower cognitive function compared with non-users [52]. The aforementioned explanations for a lack of association between TDB and physical function may be equally applicable to the lack of association between TDB and cognitive function. That is, drug prescribing may be more optimal for patients with dementia compared with healthy older populations because of a higher emphasis on tolerability and quality of life instead of treatment of symptoms and quantity of life. Alternatively, the effects of drug use on cognitive function may be harder to detect in patients with dementia due to the large variety of dementia-related influencing factors.

Similar to TDB, we found no evidence in support of our hypothesis that higher AChDBI and SDBI are separately associated with lower cognitive function. In older women, higher anticholinergic burden (not measured with DBI) correlated more strongly with lower global cognitive function than sedative burden [58]. No evidence was reported for different cognitive domains. In addition, a higher AChDBI was associated with lower memory performance and lower performance on the Trail Making Test B in cognitively healthy older adults [25]. No associations between SDBI and cognitive domains were reported. The lack of discernible associations of anticholinergic versus sedative drugs on cognitive function in our study may result from the simultaneous dysregulation of the excitatory/inhibitory systems by either anticholinergic or sedative drugs in patients with dementia, as described previously in this paper.

### Study limitations

Several limitations warrant caution in interpreting our results. First, the sample size of the current study is small compared to other studies [18] and the studied DBI subgroups were of unequal sizes. This might have negatively affected statistical power. Second, our sample consisted of patients with mild to moderate dementia, with the mean MMSE score indicating moderate dementia. Therefore, the results are not directly generalizable to a more severe dementia population. Also, due to the cross-sectional design of our study, we cannot exclude the issue of confounding by indication. We are thus unable to make claims about causal effects of DBI drugs on functional outcomes in dementia. A complicating factor is that the efficacy of DBI drugs in the later stages of dementia is not yet adequately assessed. To gain an optimal understanding of the positive and negative effects of DBI drugs in dementia patients, future experimental studies on the efficacy of DBI drugs with varying degrees of dementia are needed. Additionally, differences between how previous studies and we classified DBI is one source of inconsistency [18]. The source of this inconsistency is a lack of consensus as to which drugs to classify as anticholinergic, sedative, or both. In particular, the current DBI may be an underestimation of the true anticholinergic and sedative burden, as drugs with both anticholinergic and sedative effects are classified as anticholinergic only. Thus, our indices, compared with other studies, could yield a different drug burden value compared with other methods. To minimize such differences, we used a detailed classification process that included several reliable sources and we also strictly adhered to the original method [15] in estimating DBI. Also, the DBI does not account for medications as needed, which could have influenced the study results if participants took such medications before the assessments. Furthermore, patients with dementia in NHs may be inherently different from community-dwelling patients. Associations of DBI with functional outcomes are therefore not directly generalizable to a community-dwelling dementia population. Further research could focus on the associations of DBI with functional outcomes in different samples of community-dwelling versus institutionalized patients with dementia. Longitudinal research could be done by following dementia patients through the process of institutionalization, while tracking medication records and functional outcomes. In addition, the mean number of comorbidities in our sample (*M* = 2) was lower compared with that in another sample of NH patients with dementia (*M* = 4, on a summary scale (not FCI)) [61]. The difference between our study and the study by Sloane et al. [61] could result from the exclusion of people with multiple health-related conditions in our sample, which could have resulted in a healthier-than-average group of participants. Contrarily, the FCI does not comprise all medical conditions, and the lower comorbidity scores in our sample may have resulted from the exclusion of several medical conditions on the FCI, such as cancer or thyroid disease. Altogether, caution is advised when generalizing our results to other NH patients with dementia. Moreover, the DBI does not include a weighting factor for the relative anticholinergic activity of each DBI drug. Not all drugs have equally strong anticholinergic effects [62]. The use of specifically high potency anticholinergics has been linked to an increased risk of all-cause dementia in older adults [63]. Duran et al. [64] provide a differentiation in anticholinergic potency of drugs with anticholinergic properties (Appendix 2). Appendix 2 shows that our sample predominantly used drugs with low anticholinergic potency, which is not reflected in the DBI. This may at least partly account for the absence of a relationship between DBI and functional outcomes and warrants a careful generalization of our results to other NH populations with dementia. Lastly, Dutch NHs might be inherently different in terms of drug-prescribing practices compared with NHs in other countries [65]. Therefore, we urge caution in generalization of these results to other NHs.

### Clinical implications

A lack of association between DBI and functional outcomes raises questions about the DBI as a clinical assessment tool of drug burden in patients with dementia. DBI is considered as a valid and useful tool to evaluate drug burden in many populations. However, it does not account for the many drug-drug interactions between DBI drugs. Also, the DBI does not consider possible adverse effects of other non-DBI-contributing drugs. The identification of inappropriate prescribing in patients with dementia is particularly challenging because evidence-based guidelines are lacking and health care practitioners are unsure about the best prescribing choices [66]. As a result, DBI might over- or underestimate true drug burden. To prevent underestimation of drug burden, perceived medication effects could be assessed by inquiring patients and caregivers.

## Conclusion

In contrast with previous studies in healthy older adults, DBI did not correlate with cognitive and physical functions in a sample of institutionalized patients with dementia. The lower use of DBI contributing drugs in our sample compared with a community-dwelling healthy population might indicate that drug prescribing is more optimal for patients with dementia compared with cognitively healthy older adults. Further experimental research into the efficacy of DBI drugs for patients with dementia of different severities and etiologies, and in different care settings, is needed to clarify the relationship between DBI and functional outcomes in patients with dementia. To achieve or maintain optimal disease management for patients with dementia, prudence is urged when prescribing anticholinergic or sedative drugs for the treatment of neuropsychiatric complaints.

## Electronic supplementary material

Appendix (DOCX 20 kb)
