# Supplementary material for: Relationship between drug burden and physical and cognitive functions in a sample of nursing home patients with dementia
Source: Eur J Clin Pharmacol. 2017 Sep 18;73(12):1633–42. doi: 10.1007/s00228-017-2319-y (PMC5684292; doi:10.1007/s00228-017-2319-y)
Supplement: Supplementary file 1 — (DOCX 20 kb) [file 228_2017_2319_MOESM1_ESM.docx]

*Appendix 1. Exclusion criteria and recruitment procedures for the sample*

Exclusion criteria

Exclusion criteria for the current sample were: being wheelchair bound, presence of severe cardiovascular problems that limit physical activity, serious visual or auditory problems, history of alcoholism, personality disorders, cerebral trauma, hydrocephalus, neoplasm, disturbances of consciousness or focal brain disorders.

Recruitment procedures

To recruit the participants, the investigator contacted nursing home facilities and provided information letters and informed consent documents. Representatives of the nursing home facilities screened potential participants and provided them with the information and informed consent documents. After informed consent was obtained, potential participants were screened for eligibility by the investigator. Potential participants were included in the study when they were found to be eligible after screening by the investigator.

*Appendix 2. DBI contributing drugs in the study sample*

| **ATC-code** | **Generic name** | **Classification anticholinergic (A) / sedative (S)** | **Anticholinergic potency** | **Recommended minimum daily dose* (mg)** | **n (*n* total = 67)** |
| --- | --- | --- | --- | --- | --- |
| G04BD04 | Oxybutynine | A | high | 10.0 mg | 1 |
| G04BD07 | Tolterodine | A | high | 4.0 mg | 1 |
| G04BD08 | Solifenacine | A | undetermined | 5.0 mg | 2 |
| G04BD11 | Fesoterodine | A | undetermined | 4.0 mg | 1 |
| N02AB03 | Fentanyl | A | low | 0.05 mg | 1 |
| N03AF01 | Carbamazepine | A | low | 200.0 mg | 2 |
| N04BA02 | Levodopa/carbidopa | S | low | 150.0 mg | 1 |
| N05AF05 | Zuclopentixol | A | undetermined | 10.0 mg | 1 |
| N05AH02 | Clozapine | A | high | 25.0 mg | 1 |
| N05AH03 | Olanzapine | A | low | 5.0 mg | 1 |
| N05AH04 | Quetiapine | A | low | 200.0 mg | 3 |
| N05AX08 | Risperidon | A | low | 1.0 mg | 6 |
| N06AA09 | Amitriptyline | A | high | 10.0 mg | 6 |
| N06AA10 | Nortriptyline | A | high | 10.0 mg | 4 |
| N06AB03 | Fluoxetine | A | low | 20.0 mg | 1 |
| N06AB04 | Citalopram | A | low | 10.0 mg | 16 |
| N06AB05 | Paroxetine | A | low | 20.0 mg | 4 |
| N06AX16 | Venlafaxine | S | low | 75.0 mg | 2 |
| M03BX01 | Baclofen | A | low | 30 mg | 1 |
| N03AE01 | Clonazepam | A | low | 2.0 mg | 2 |
| N03AX14 | Levetiracetam | S | undetermined | 500.0 mg | 2 |
| N05AD05 | Pipamperon | S | undetermined | 20.0 mg | 6 |
| N05BA01 | Diazepam | A/S^a^ | low | 2.0 mg | 1 |
| N05BA04 | Oxazepam | S | low | 7.5 mg | 12 |
| N05BA05 | Clorazepinezuur | S | high/low^b^ | 5.0 mg | 1 |
| N05BA06 | Lorazepam | S | low | 0.5 mg | 2 |
| N05BA12 | Alprazolam | S | high/low^b^ | 0.5 mg | 1 |
| N05CD02 | Nitrazepam | S | undetermined | 2.5 mg | 2 |
| N05CD06 | Lormetazepam | S | undetermined | 0.5 mg | 1 |
| N05CD07 | Temazepam | A/S^a^ | low | 10.0 mg | 12 |
| N05CF01 | Zopiclon | S | low | 3.75 mg | 2 |
| N05CF02 | Zolpidem | S | low | 5.0 mg | 2 |
| N06AX11 | Mirtazapine | A | low | 15.0 mg | 3 |
| N07CA02 | Cinnarizine | S | undetermined | 25.0 mg | 2 |

**minimum oral dose. ^a^Drug has both anticholinergic and sedative properties, but is classified as anticholinergic in the DBI. Duran et al. [64] was used to determine anticholinergic potency. ^b^Strong discrepancy [64].*

*Appendix 3. Functional Comorbidity Index*

| **1** | Arthritis (rheumatoid and osteoarthritis) |
| --- | --- |
| **2** | Osteoporosis |
| **3** | Asthma |
| **4** | Chronic obstructive pulmonary disease (COPD), acquired respiratory distress syndrome (ARDS), or emphysema |
| **5** | Angina |
| **6** | Congestive heart failure (or heart disease) |
| **7** | Heart attack (myocardial infarct) |
| **8** | Neurological disease (such as multiple sclerosis or Parkinson’s) |
| **9** | Stroke or TIA |
| **10** | Peripheral vascular disease |
| **11** | Diabetes types I and II |
| **12** | Upper gastrointestinal disease (ulcer, hernia, reflux) |
| **13** | Depression |
| **14** | Anxiety or panic disorders |
| **15** | Visual impairment (such as cataracts, glaucoma, macular degeneration) |
| **16** | Hearing Impairment (very hard of hearing, even with hearing aids) |
| **17** | Degenerative disc disease (back disease, spinal stenosis, or severe chronic back pain) |
| **18** | Obesity and/or body mass index >30 (weight in kg/height in meters^2^) |
